# An optimized workflow for MS-based quantitative proteomics of challenging clinical bronchoalveolar lavage fluid (BALF) samples

**DOI:** 10.1186/s12014-023-09404-1

**Published:** 2023-04-02

**Authors:** Danielle O. Weise, Monica E. Kruk, LeeAnn Higgins, Todd W. Markowski, Pratik D. Jagtap, Subina Mehta, Alan Mickelson, Laurie L. Parker, Christine H. Wendt, Timothy J. Griffin

**Affiliations:** 1 Division of Pulmonary, Allergy, Critical Care and Sleep Medicine, Medical School, University of Minnesota, Minneapolis, MN USA; 2 Department of Biochemistry, Molecular Biology and Biophysics, University of Minnesota, Minneapolis, MN USA; 3 Minneapolis VA Health Care System, Minneapolis, MN USA

**Keywords:** Bronchoalveolar lavage fluid, BALF, Quantitative proteomics, Mass spectrometry, Sample preparation, Lung disease

## Abstract

**Background:**

Clinical bronchoalveolar lavage fluid (BALF) samples are rich in biomolecules, including proteins, and useful for molecular studies of lung health and disease. However, mass spectrometry (MS)-based proteomic analysis of BALF is challenged by the dynamic range of protein abundance, and potential for interfering contaminants. A robust, MS-based proteomics compatible sample preparation workflow for BALF samples, including those of small and large volume, would be useful for many researchers.

**Results:**

We have developed a workflow that combines high abundance protein depletion, protein trapping, clean-up, and in-situ tryptic digestion, that is compatible with either qualitative or quantitative MS-based proteomic analysis. The workflow includes a value-added collection of endogenous peptides for peptidomic analysis of BALF samples, if desired, as well as amenability to offline semi-preparative or microscale fractionation of complex peptide mixtures prior to LC–MS/MS analysis, for increased depth of analysis. We demonstrate the effectiveness of this workflow on BALF samples collected from COPD patients, including for smaller sample volumes of 1–5 mL that are commonly available from the clinic. We also demonstrate the repeatability of the workflow as an indicator of its utility for quantitative proteomic studies.

**Conclusions:**

Overall, our described workflow consistently provided high quality proteins and tryptic peptides for MS analysis. It should enable researchers to apply MS-based proteomics to a wide-variety of studies focused on BALF clinical specimens.

**Supplementary Information:**

The online version contains supplementary material available at 10.1186/s12014-023-09404-1

## Introduction

### BALF: A valuable clinical sample for studying lung disease

Bronchoalveolar lavage fluid (BALF) is a clinical sample, generally collected from patients with lung conditions, via a bronchoscopy passed through the upper airway to the lower airways of the lung. A saline solution is introduced, effectively lavaging the distal lung tissue followed by aspirating the fluid or lavage, providing a means to obtain cells and sample the alveolar lining fluid and its molecules for further analysis. BALF’s value for molecular characterization of lung disease has been long known [1] and described for various clinical investigations [2–4].

The value of BALF for investigation of lung disease and biology stems from its rich repertoire of biomolecules, many of which are lung specific. The distal lung sampled in BALF collection contains alveolar macrophages, and, in disease, various inflammatory cells, along with cell secreted molecules (proteins and metabolites), host and microbial DNA [5], RNA (primarily packaged in extracellular vesicles [6]) and lipids [7]. Proteins have long been known to be a major component of BALF [8], including proteins present in high amounts specific to disease pathologies (e.g. mucins, surfactant proteins) [9, 10]. The fluid also contains endogenous peptides generated as products of protease activity on larger proteins, some of which have biological activity and can serve as biomarkers [11, 12]. Small molecule metabolites, acted upon by enzymes, are also detectable [13–15]. Finally, extracellular vesicles, packed with nucleic acids, proteins and other molecule types have also been well-described [16, 17].

### BALF MS-based proteomics: history and existing challenges

Given the prominence of proteins within BALF, much attention has been given to applying mass spectrometry (MS)-based proteomics methods to the characterization of these samples. Early applications focused on using two-dimensional gel electrophoresis to separate and visualize complex protein mixtures derived from BALF [8], followed by digestion of separated proteins with trypsin and analysis using nanoscale liquid chromatography (LC) tandem mass spectrometry (MS/MS) to collect mass spectra of fragmented peptides for subsequence sequence database searching, peptide identification and protein inference [18]. Over the last two decades numerous studies of BALF collected from patients with diverse lung conditions have been described in the literature [4, 19–21].

From the outset, the challenges that BALF presents to MS-based proteomic analysis has been appreciated and described in numerous publications [22], including a recent report from members of the American Thoracic Society [23]. As outlined in these publications, the analytical challenges to working with BALF are numerous. These stem from the inherent chemical complexity of lung tissue exudate that makes up BALF, which presents these challenges: (1) presence of many plasma-derived, high abundance proteins (e.g. albumin, transferrin, etc.) that may suppress detection of lower abundant, lung tissue-derived proteins; (2) potential for interfering molecules found in mucus, and/or lipid surfactants, as well as high salt content from saline used in the BALF collection, all of which are generally not compatible with nanoscale LC–MS/MS based proteomic systems; (3) dilution of tissue-derived molecules due to large volumes of saline that is sometimes used to collect BALF samples; and (4) limiting amounts of protein material collected, depending on the subject (e.g. children vs adults) and/or disease pathology being studied, challenging deep detection of proteins.

Given these challenges, it is not surprising that a number of publications have described workflows for MS-based proteomic analysis of BALF over the prior two or more decades [24–26]. As MS-based technologies have improved in their sensitivity and accuracy for qualitative and quantitative proteomics, the depth of BALF proteomic studies has also increased. Whereas early efforts using MS-based proteomics which could only identify tens to hundreds of proteins [8], newer conceptions have greatly expanded the detectable proteins within these complex samples.

Notably, a recent qualitative study using a contemporary high-resolution LC–MS/MS system and employing depletion of high abundance plasma proteins coupled with extensive, semi-preparative offline high pH HPLC fractionation has identified over 4000 proteins in BALF from lung cancer patients [27]. A recent quantitative study in BALF used semi-preparative offline high pH HPLC fractionation and label-free quantification to identify several thousand proteins, including those differentially expressed in diseases related to lung connective tissue [28]. Another recent study showed the potential for emerging data-independent acquisition (DIA) to quantify BALF proteins in lung cancer, where direct LC–MS analysis of tryptic digests from patient samples quantified over 600 proteins in these samples [29].

Despite their success, the workflows employed for preparing samples in these studies have some limitations that prevent broader adoption for BALF studies. Notably, those studies demonstrating the deep identification of thousands of BALF proteins utilized relatively large amounts of starting BALF sample – 20 mL or more of individual samples [28] or pooling patient samples to generate tens of milliliters of sample [27] in order to yield protein amounts necessary for semi-preparative scale fractionation and LC–MS/MS analysis. Although these amounts of starting material are acceptable for small scale proof-of-concept studies, in many cases BALF sample volumes available for analysis are only in the low milliliter range, especially for studies involving children. The methods also utilize processing steps such as precipitation geared towards higher amounts of total protein in order to remove contaminants, which may be susceptible to sample loss in samples with lower amounts of material. Finally, these studies have not demonstrated their compatibility with contemporary quantitative proteomic methods geared towards analysis of larger cohorts of patients (e.g. highly multiplexed isobaric peptide labeling), which is necessary for large-scale studies investigating clinical BALF samples. Therefore, a need still exists for a robust sample processing workflow amenable to BALF samples collected in low milliliter amounts, offering the ability to sensitively detect even lower abundant lung proteins, and be applied to multiplexed quantitative analysis of larger patient cohorts.

### A robust and novel workflow for quantitative proteomics of BALF

Here, we describe a robust sample processing workflow with flexibility to a wide variety of studies focused on the proteomic characterization of BALF, addressing for the first time many of the challenges encountered in MS-based proteomic analysis of these challenging clinical samples. The workflow brings together high abundance protein depletion, efficient protein trapping and contaminant removal using S-Trap columns, and compatibility to multiplexed isobaric peptide tagging of resulting trypsin digested protein samples. The workflow includes a value-added step for collecting endogenous peptides for MS-based peptidomics, if desired, while being amenable to a wide range of total protein yields (tens of micrograms down to a microgram or less) resulting from varying amounts of available BALF volumes. The workflow utilizes offline high pH HPLC peptide fractionation compatible with this range of protein yields (semi-preparative scale for large yields, microscale for limited yields). Through the analysis of clinically derived samples from patients with Chronic Obstructive Pulmonary Disease (COPD), we demonstrate the repeatability of our workflow using several common approaches for protein quantification, as well as the ability to process a large number of clinical samples, indicating its utility for quantitative MS-based proteomics of BALF. We also demonstrate the ability of this processing method to yield ample amounts of protein when applied to a relatively large cohort of 45 clinical BALF samples. Our workflow should be of value for a wide range of researchers seeking to understand proteome dynamics related to lung health and disease in routinely collected clinical BALF samples.

## Results

**Fig. 1:**
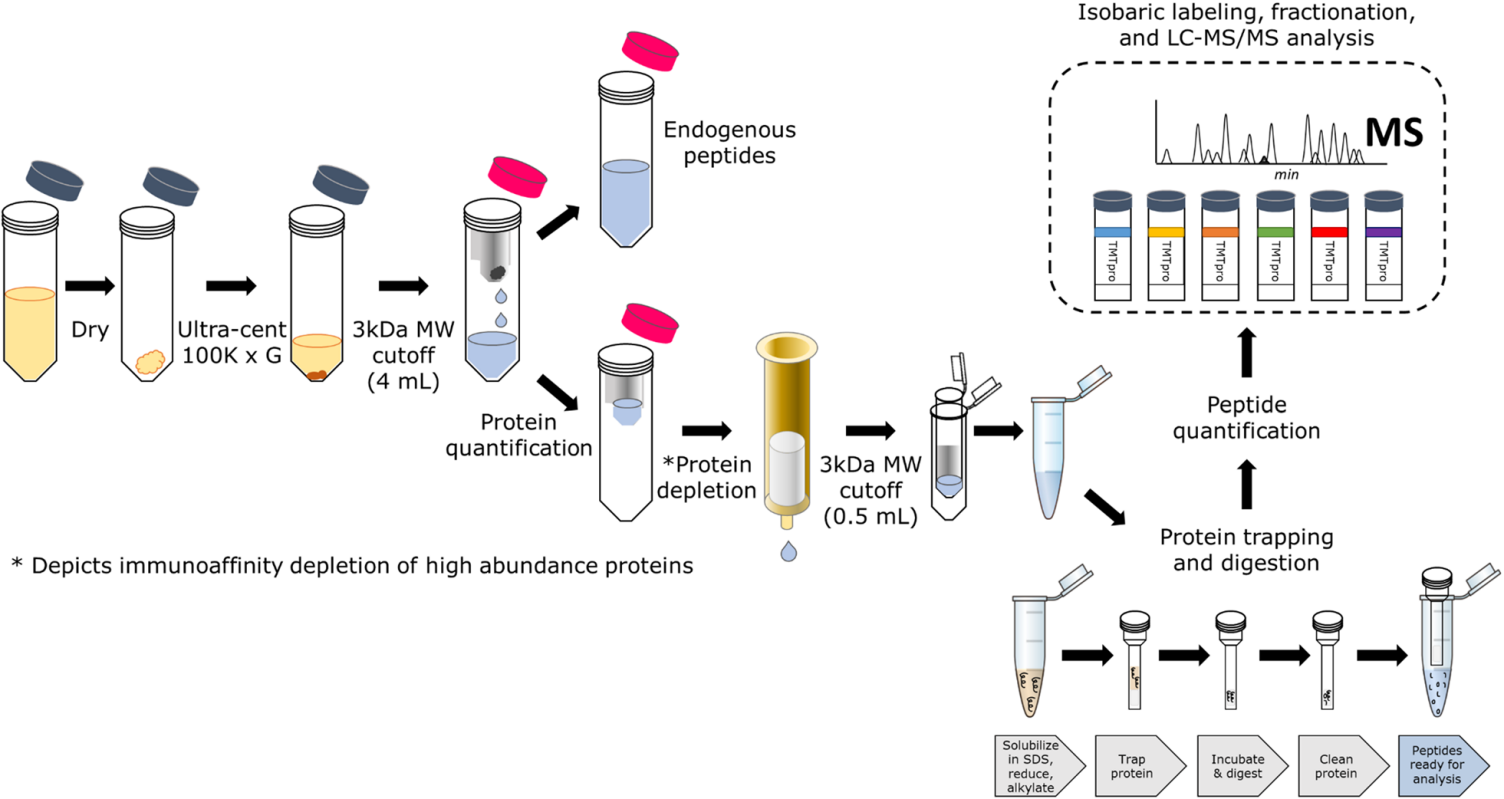
Key steps of BALF processing workflow for quantitative LC–MS/MS analysis. The workflow includes value-added collection of endogenous peptides for analysis (if desired), along with immunoaffinity-based depletion of the 14 most abundant plasma proteins, as well as protein trapping for concentration, clean-up, and tryptic digestion. The resulting peptides are compatible with isobaric peptide labeling or label-free analysis by LC–MS/MS

Figure 1 shows the BALF processing workflow, compatible with LC–MS/MS and quantitative proteomic analysis. Details of each step are provided in the “Methods” section. The key steps to the workflow include using a molecular weight (MW) cutoff spin filter which simultaneously concentrates the higher MW proteins and enables collection of endogenous peptides for analysis by LC–MS, if desired. Concentrated proteins are subjected to immunoaffinity depletion of high abundance plasma proteins, followed by concentration of non-retained proteins, and cleanup and trypsin digestion using protein trapping. Peptide mixtures are then ready for isobaric labeling, if desired, and/or direct analysis using nanoscale LC–MS/MS.

Initially our protocol included a protein precipitation step, similar to other past protocols [28, 30], to concentrate, and ostensibly eliminate contaminants from the BALF proteins. Methanol:chloroform precipitation was chosen as a means to phase separate potential lipid or surfactant contaminants from soluble proteins [30]. However, we found that the precipitation step was inconsistent in its effectiveness at removing contaminants, as in some cases downstream LC–MS/MS analysis showed contaminants that obscured detection of BALF-derived peptides (see Additional file 1: Figure S1 for representative contamination results). Precipitation also had the downside of expanding sample volumes, which required extra handling and concentration steps.

As a solution, we introduced a concentration step using a MW cutoff spin filter, and downstream protein trapping via the S-Trap technology [31, 32]. In our hands, protein trapping provided a means to efficiently capture proteins, remove contaminants, concentrate samples, and conduct tryptic digestion all within the same disposable filter, while providing an easy means to collect the peptides for further processing and/or analysis. After implementing the protein trapping step, we eliminated problems with contaminants.

Our workflow also provides a means to enrich endogenous peptides from BALF. These are most likely peptides processed proteolytically and have been shown to have potential diagnostic value in BALF samples [31, 32]. We have found that the flow-through collected from the 3 kDa molecular weight cut-off filters using starting volumes of 1–5 mL of BALF contain micrograms of endogenous peptides, which can be concentrated and desalted using STAGE Tips and detected directly by LC–MS/MS. Indeed, in an ongoing study applying our processing workflow to a larger cohort of 45 individual COPD patient samples, we found that we recovered an average of 37.9 µg of peptides from these samples using this peptide enrichment step, ample amounts for analysis by LC–MS/MS (see Additional file 2: Table S1 which shows endogenous peptides recovered across these patient BALF samples).

The yield of high-quality proteins, and resulting tryptic peptides, is another critical parameter for ensuring deep and accurate results in MS-based proteomics from challenging clinical samples such as BALF. Once we had determined the ability of our workflow to reliably eliminate contaminants from BALF samples, we assessed the yield of proteins and tryptic peptides from starting sample amounts representative of those generated from patients in the clinic. Table 1 shows a summary of results from 45 processed BALF samples collected from COPD case and control patients, each with an average starting volume of about 4 mL, which represents an amount of clinical sample that is commonly collected from infants and children where volumes are proportional to body weight [33, 34]. Here, we show the average amounts of protein, and peptides after digestion with trypsin, quantified at key points across the steps shown in Fig. 1. Important values shown in Table 1 include the amount of protein available after depletion of high abundance proteins (including a calculation of % depletion), and also the final amount of peptides available after protein digestion with trypsin and elution from the S-Trap. Additional file 2: Table S1 shows the values for the 45 individual samples used for this assessment. Although there is some variation in these numbers depending on the sample, the processing workflow consistently yields microgram amounts of high-quality tryptic peptides, ample amounts for deep proteomic analysis using contemporary LC–MS/MS instrumentation platforms.

**Table 1:** Yield of proteins and peptides from 45 individual BALF samples

Average μg recovered after ultra/3 kDa cutoffs	Average μg loaded for depletion	Average μg recovered after depletion	Average % depletion	Average μg loaded into S-Traps	Average μg of recovered clean peptides
261.8 ± 191.9	234.7 ± 131.7	12.3 ± 15.0	94.5 ± 5.3	7.1 ± 2.9	5.1 ± 2.7

**Fig. 2:**
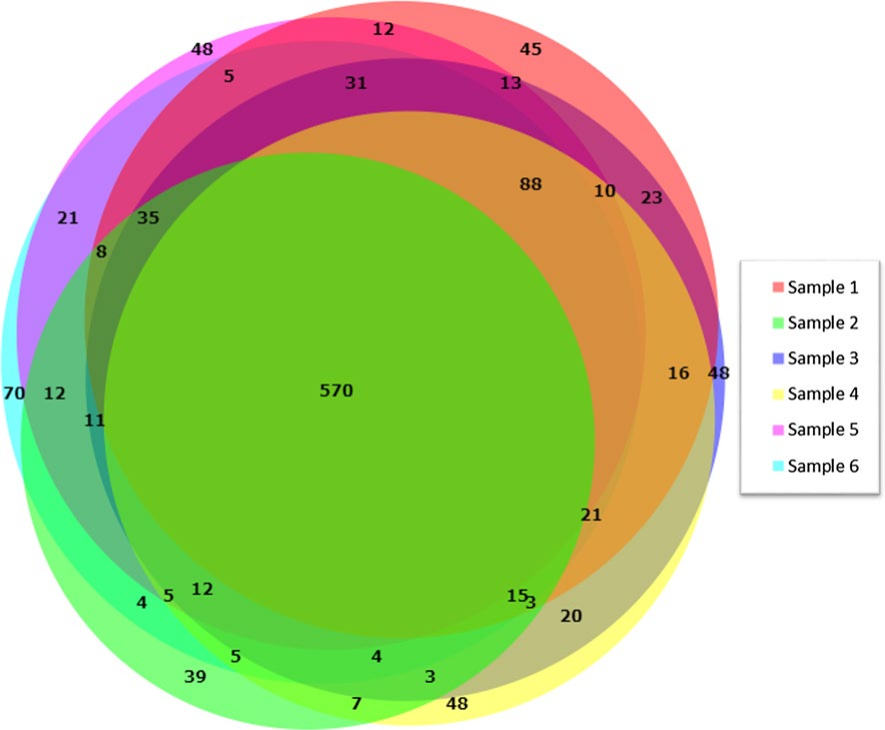
Venn diagram of proteins identified from LC–MS/MS analysis of six representative BALF samples processed via our workflow.

We also assessed the depth of results from a direct LC–MS/MS analysis, selecting six representative BALF samples for processing, followed by analysis using an Orbitrap Eclipse system. Figure 2 shows a Venn diagram of the proteins identified from six of these samples, which yielded an average of 988 proteins identified via direct LC–MS/MS analysis. Parameters were set at 1% FDR with 2 or more unique peptides for protein identification and grouping (see Methods for details).

The amenability of any sample processing workflow to quantitative analysis is critically important, as most researchers will seek to compare changes in BALF protein abundance between different conditions. As such, we carried out a repeatability study using representative patient BALF samples using isobaric Tandem Mass Tag (TMT) reagents for multiplexed quantitative analysis [35]. Here, two starting BALF samples (called here Test 1 and Test 2) were divided into two equal parts and each portion taken through the protein depletion and clean-up steps of the workflow. After tryptic digestion, the peptides from these test samples (5 µg per sample) were labeled with TMT reagents, as part of a larger testing experiment using the 16-plex TMTPro labeling kit, followed by fractionation using offline high pH HPLC.

Table 2 shows results from the two replicated TMT-labeled BALF samples (Test 1 and Test 2). We found that samples divided into equal amounts prior to protein depletion had average protein ratios close to the expected value of 1, with acceptable CVs for quantitative MS-based proteomics [36] demonstrating repeatability of our workflow. In terms of qualitative results, across the entire 16-plex TMT experiment, we identified with high confidence 1844 protein groups from 24,306 peptides. Additional file 3: Table S2 shows the complete set of protein identification results from this TMT-based experiment, and the quantitative analysis of the Test 1 and Test 2 replicate samples.

**Table 2:** Results from a TMT-based quantitative repeatability experiment in BALF samples using our sample preparation workflow

Peptides identifed (1% FDR)	Protein groups (1% FDR)	Average replicate TMT abundance ratios
20,399	1816	0.92 ± 0.27 (29.6% CV)

To further prove the repeatability of our workflow, we selected a single BALF patient sample and split it into three equal portions. Each portion was separately processed using our workflow, and results were analyzed with both label free quantification (LFQ) and spectral counting. For LFQ analysis, even when using a relatively low threshold of two or more unique peptides to quantify proteins, average abundance ratios across replicates measured by LFQ were 0.89, with average coefficients of variation (CVs) of 29.4%. When considering more stringent thresholds of 4 peptides per protein for quantification, the average LFQ CVs fell to 23.7%. For quantification using normalized spectral counting, we measured the average abundance ratio across replicates to be 1.03 with average CV of 28.3%. Additional file 4: Table S3 shows the identified peptides and proteins from this experiment, and the LFQ and spectral counting quantification results from the replicate samples.

Finally, we also tested the effectiveness of handling samples that yield lower amounts of protein prior to LC–MS/MS analysis. In some cases, individual BALF samples yielded very low amounts of recovered digested peptides, yielding only about 1 µg. To demonstrate the amenability of our workflow to in-depth, quantitative analysis using TMT labeling even at these low amounts of protein yields, we adapted a microscale labeling [37] and fractionation method [38, 39]. Here, to minimize sample handling steps the peptides are labeled while bound to C18 stationary phase. In this way, each of the 16 separate samples with starting peptide amounts of 1 µg each were subjected to TMT labeling, followed by microscale high pH fractionation (referred to here as “microfractionation”) resulting in 9 fractions for LC–MS/MS analysis. In total, we analyzed three different 16-plex TMT labeled groups of samples (48 total TMT-labeled samples) from our larger cohort of clinical BALF samples and analyzed them either with or without microfractionation. Table 3 shows the quantitative results of these experiments, showing average peptides and proteins identified across these different groups. Microfractionation increased the depth of protein identification significantly, by approximately twofold. Additional file 5: Table S4 shows the number of proteins identified within one of these 16-plex TMT labeled group of samples.

**Table 3:** Amenability of the workflow to material-limited samples and the effects of microfractionation on sampling depth. Averages for peptides and proteins identified across three 16-plex TMT groups are shown

No fractionation		With microfractionation	
Peptides identified (1% FDR)	Protein groups (1% FDR)	Peptides identified (1% FDR)	Protein groups (1% FDR)
4770 ± 1726	524 ± 77	9902 ± 933	1127 ± 57

## Discussion

Our results demonstrate the effectiveness of our workflow for preparing samples for a wide variety of proteomic studies in BALF samples. BALF is a commonly collected clinical specimen, rich in biomolecules, and valuable for molecular characterization of lung disease and health. Proteins are a main component of BALF [9, 10, 21] making this sample type a prime target for MS-based proteomic analysis. However, the small sample volume, potential for contaminants, and suppression by high abundance proteins is known to limit many researchers in their attempts to analyze BALF [23].

Our workflow offers several significant advantages that overcome these limitations, and in doing so demonstrates the novelty of this BALF sample preparation method compared to those described previously. These demonstrated advantages include:

- The streamlined sample handling steps are amenable to relatively small starting volumes of BALF (as low as 1 mL of starting volume), in contrast to other studies that have described analysis of tens of milliliters of starting samples [27]; we identify similar numbers of proteins as these past studies [27], with far less starting material. We also demonstrate the use of our workflow on a large cohort of 45 individual BALF patient samples, where we recovered ample amounts of tryptically-digested peptides from all of these samples for deep MS-based proteomic analysis.
- The high-quality peptide mixtures generated by the workflow are amenable to either semi-preparative offline high pH HPLC fractionation (for sample amounts producing tens of micrograms of peptides) or microfractionation (for samples producing as little as 1 µg of peptides). This is the first such demonstration of a workflow for BALF processing that utilizes fractionation on such small amounts of peptide yields to improve depth of sampling.
- The processing steps, including depletion of high abundance proteins, S-Trap based purification and tryptic digestion, are quantitatively repeatable, with accuracy and precision at levels as good, if not better, than those expected from non-targeted MS-based quantitative proteomics analyses [36], making this workflow amenable to downstream quantitative proteomics.
- We demonstrate the use of multiplexed TMT-labeling with our workflow as an example quantitative method, and also label-free methods (LFQ and spectral counting); our results indicate that the workflow should also be amenable to emerging data independent acquisition (DIA) methods [40].
- Our approach offers a unique, value-added, easy enrichment of endogenous peptides from BALF samples using molecular weight cutoff spin filters; these peptides are amenable to direct analysis using LC–MS/MS and offer additional information on diagnostic signatures and/or proteolytic processing.

During the course of our work, it came to our attention that our high abundance depletion spin columns (Seppro, Sigma Aldrich) were discontinued for manufacturing. Fortunately, a very similar product is available from Thermo Fisher (High-Select Top14 Abundant Protein Depletion Resin) which can be used in a similar spin-column format to the methods we describe in our workflow. This product targets many of the same proteins as the Seppro product and should be easily implemented within our general workflow.

## Conclusions

We have demonstrated a robust sample preparation workflow for clinical BALF samples. This workflow provides high quality proteins, and resulting tryptic peptides, for analysis in contemporary MS-based proteomics instrument platforms. Although we demonstrate its effectiveness in starting sample amounts down to 1 mL in volume, further optimization may be necessary for very dilute clinical samples or those with very small total volumes well under 1 mL, such as those collected from infants or small children [33, 34]. Nevertheless, our workflow should be effective on the vast majority of BALF samples collected in the clinic using standardized methods, and useful for studying proteome dynamics in a wide variety of studies focused on lung health and disease.

## Methods

### BALF collection

BALF was obtained using standard procedures [41, 42]. Sample collection was performed by sequentially instilling and then withdrawing 50 mL aliquots of sterile normal saline up to a total of 200 mL into the right middle lobe or lingula. Samples were centrifuged to remove cells, aliquoted and immediately stored at -80 °C prior to processing and underwent one freeze–thaw cycle.

### Initial BALF processing

BALF samples were thawed, vortexed, and centrifuged at 500 × G for 10 min at 4 °C. The supernatant was transferred to a new 15 mL conical tube. The insoluble pellet was suspended in 500 uL of PBS and stored at − 70 °C for future use. BALF supernatant was refrozen prior to drying through lyophilization. Samples were resuspended in 1 mL of LC–MS grade water, ultra-centrifuged at 100,000 × G for 1 h, and the supernatant was removed for further processing. The remaining pellet was suspended in 200 uL of PBS and added to the soft spin pellet collected prior to lyophilization.

### Molecular weight cutoff step to collect endogenous peptides

Amicon Ultra-4 centrifugal filters, MWCO of 3 kDa, were conditioned with 4 mL of 5% methanol in LC–MS grade water and an additional rinse of 4 mL LC–MS grade water. BALF supernatant was ultra-centrifuged at 4,000 × G for 1 h at 4 °C. Flow-through, containing endogenous peptides, was frozen and stored at −70 °C for future peptide analysis. The concentrated protein was removed from the filter’s sample reservoir and transferred to a 2.0 mL LoBind tube.

### Quantification of proteins with BCA

BALF proteins were quantified using the Pierce BCA protein assay. BSA standards and samples were analyzed in a microplate reader at an absorbance of 562 nm. A standard curve was calculated to determine protein amounts in BALF samples.

### High abundance protein depletion

Seppro IgY14 spin columns were used to remove fourteen highly abundant plasma proteins (Albumin, IgG, α1-Antitrypsin, IgA, IgM, Transferrin, Haptoglobin, α2-Macroglobulin, Fibrinogen, Complement C3, α1-Acid Glycoprotein, HDL, LDL) from BALF samples, leaving an enriched pool of low abundance proteins. Prior to sample loading, spin columns were washed with two blank samples of Seppro dilution buffer to remove non-covalently bound IgY from the beads. (Each “wash” included: addition of buffer to spin columns, mixing of the beads by mechanical inversion/shaking of the column, and centrifugation at 400 × G for 30 s). Known amounts of BALF protein in 1 × Seppro dilution buffer (Tris-buffered saline), were loaded into the spin column. Samples were mixed for 15 min, centrifuged at 400 × G for 30 s, and flow-through was collected. An additional wash of the column with 1 × Seppro dilution buffer was performed and a second flow through was collected. The two BALF washes were kept on ice for further analysis. Spin columns were washed twice with 1 × Seppro dilution buffer. Bound proteins were stripped from the beads with four washes of 1 × Seppro glycine-based stripping buffer, and immediately neutralized with 1 × Seppro neutralization buffer (Tris (hydroxymethyl) aminomethane). Beads were resuspended in a 1 × Seppro dilution buffer containing 0.02% sodium azide, and stored at 4 °C.

### Molecular weight cutoff to concentrate the two washes after Seppro

Amicon Ultra-0.5 mL centrifugal filters, MWCO of 3 kDa, were conditioned with 0.5 mL of 5% methanol in LC–MS grade water and an additional rinse of 0.5 mL LC–MS grade water. The initial BALF flow-through collected from Seppro was ultra-centrifuged at 14,000 × G for 1 h at 4 °C. The second BALF flow-through was added and ultra-centrifuged at 14,000 × G for 1 h at 4 °C. Concentrated protein was removed from the filter’s sample reservoir and transferred to a 1.5 mL LoBind tube.

### Quantification of proteins with BCA

BALF proteins were quantified using the Pierce BCA protein assay. BSA standards and samples were analyzed via Nanodrop at an absorbance of 562 nm. A standard curve was calculated to determine protein amounts in BALF samples.

### Protein trapping, clean-up and tryptic digestion

Post-depletion BALF protein was frozen and lyophilized in a speed vac. Dried BALF samples were solubilized in 5% SDS, 50 mM TEAB, pH 8.5, sonicated at 90 sonics for 5 min, and centrifuged at 12,000 × G for 8 min. The supernatant was transferred to a 1.5 mL LoBind tube. Proteins were reduced, alkylated, and acidified to pH < 1. Samples were transferred to S-Trap columns (ProtiFi) that were centrifuged at 4,000 × G for 30 s to trap proteins onto columns. Protein was washed 6 × with 100 mM TEAB in 90% LC–MS grade methanol, pH 7.55, to remove all contaminants. Trypsin Gold, MS grade (Promega), in a 1:10 ratio of enzyme to protein, was added to the S-Trap and columns were incubated overnight at 37 °C*.* Digested proteins were eluted from the column with 50% acetonitrile/50 mM TEAB, pH 8.5. BALF peptides were frozen and lyophilized in a speed vac for further use.

### Peptide assay

BALF peptides were resuspended in LC–MS grade water and quantified using the Pierce Quantitative Colorimetric Peptide Assay. Peptide digest standards and samples were analyzed via Nanodrop at an absorbance of 480 nm. A standard curve was calculated to determine peptide amounts recovered from S-Traps for each BALF sample. Samples were frozen and lyophilized in a speed vac.

### TMT labeling

#### Normal scale

For samples with higher amounts of total peptides (greater than 5 total ug), TMT16pro label reagents (Thermo Fisher) were reconstituted in anhydrous acetonitrile. A total of 5 ug of BALF protein digest for each sample was suspended in 100 mM TEAB, pH 8.5. TMTpro labels were added to BALF samples and incubated for 1 h at room temperature. 5% hydroxylamine in LC–MS grade water was added to the samples and incubated for 15 min to quench the reaction. Labeled BALF peptides were pooled, frozen, and lyophilized.

#### Microscale

For samples with lower amounts of total peptide (approximately 1 ug), TMT16pro label reagents were first reconstituted in anhydrous acetonitrile. A total of 1 ug of BALF protein digest for each sample was suspended in 0.1% formic acid in LC–MS grade water. The acidified BALF peptides were transferred to preconditioned C18 Stop and Go Extraction (STAGE) tips [43], and drawn through twice (centrifuged at 1000 × G for 1 min) to bind to the C18 stationary phase. Peptides were washed with 0.1% formic acid in LC–MS grade water and labeled with TMT16pro tags in 20 mM TEAB, pH 8 buffer. Labeled peptides were eluted with a 0.1% formic acid in 80:20 acetonitrile:water buffer followed by 20 mM ammonium formate, pH 10 in 80:20 acetonitrile:water. Each separate TMT-labeled BALF peptide sample was pooled, frozen, and lyophilized.

### Offline high pH fractionation

#### Semi-preparative scale fractionation

For normal scale, processed samples (40 ug or more total peptides after pooling) were resuspended in 50 µL of 50 mM ammonium formate and fractionated offline by high pH C18 reversed phase (RP) chromatography as described previously [44] with the following changes. A Shimadzu Prominance HPLC (Shimadzu, Columbia, MD) with a Hot Sleeve-25L Column Heater (Analytical Sales & Products, Inc., Pompton Plains, NJ) was used with a column setup of a Security Guard precolumn housing a Gemini NX C18 cartridge (Phenomemex, Torrance, CA) attached to a C18 XBridge column, 150 mm × 2.1 mm internal diameter, 5 um particle size (Waters Corporation, Milford, MA). Buffer A was 20 mM ammonium formate, pH 10 in 98:2 water:acetonitrile and buffer B was 20 mM ammonium formate, pH 10 in 10:90 water:acetonitrile. The flow rate was 200 µL/min with a gradient from 2 to 7% buffer B over 0.5 min, 7–15% buffer B over 7.5 min, 15–35% buffer B over 45 min, and 35–60% buffer B over 15 min. Fractions were collected every 2 min and UV absorbances were monitored at 215 nm and 280 nm. Peptide-containing fractions were divided into three groups, “early”, “middle”, and “late”. A volume equal to 15 milli-absorbance units of the first “early” fraction was concatenated with the first “middle” and “late” fraction, and so on. Concatenated fractions were lyophilized and cleaned with STAGE tips using Waters Oasis MCX material as the stationary phase.

#### Microscale fractionation

For microscale samples, pooled peptides (16 ug total for TMT-labeled samples) were reconstituted in 100 mM NH_4_HCO_2_, pH 10. STAGE tips were prepared with a C8 core and C18-AQ resin packed on top, and conditioned and equilibrated. Peptides were transferred to STAGE tips and drawn through twice (centrifuged at 1000 × G for 2 min) to bind them to resin/filter. Peptides were washed and eluted into fractions sequentially with increasing concentrations (5%, 7.5%, 10%, 12.5%, 15%, 17.5%, 20%, 22.5%, 25%, 27.5%, 30%, 32.5%, 35%, 40%, 50%, 60%, 70%, 80%) of acetonitrile in LC–MS grade water.

### LC–MS/MS analysis for unlabeled samples

For unlabeled peptide mixtures (e.g. no TMT labeling), we reconstituted the dried peptide fractions in 97.9:2:0.1, H2O: acetonitrile (ACN):formic acid (FA) (load solvent) and analyzed ~ 300 nanograms of each fraction by capillary LC–MS with a Thermo Fisher Scientific, Inc (Waltham, MA) Dionex UltiMate 3000 RSLCnano system on-line with an Orbitrap Eclipse mass spectrometer (Thermo Scientific, Waltham MA) with FAIMS (high-field asymmetric waveform ion mobility) separation. We injected peptides directly in load solvent and performed gradient separation on a self-packed C18 column (Dr. Maisch GmbH ReproSil-PUR 1.9 um 120 Å C18aq, 100 um ID × 40 cm length) at 55 °C with the following profile: 5% B solvent from 0 to 2 min, 8% B at 2.5 min, 21% B at 90 min, 35% B at 120 min and 90% B at 122 min with a flowrate of 400 nl/min from 0 to 2 min and 315 nl/min from 2.5 to 122 min, where solvent A was 0.1% formic acid in water and solvent B was 0.1% formic acid in ACN. The FAIMS nitrogen cooling gas setting was 5.0 L/min, the carrier gas was 4.6 L/min and the inner and outer electrodes were set to 100 °C. We scanned the CV (compensation voltage) at − 45, − 60 and − 75 for 1 s each with a data dependent acquisition method. We employed the following MS parameters: ESI voltage + 2.1 kV, ion transfer tube 275 °C; no internal calibration; Orbitrap MS1 scan 120 k resolution in profile mode from 380 to 1400 m*/z* with 50 ms injection time; 100% (4 × 10E5) automatic gain control (AGC); MS2 was triggered on precursors with 2–5 charges above 2.5E4 counts; MIPS (monoisotopic peak determination) was set to Peptide; MS2 settings (all CV’s) were: 1.6 Da quadrupole isolation window, 30% fixed collision energy, Orbitrap detection with 30 K resolution at 200 m*/z*, first mass fixed at 110 m*/z*, 54 ms max injection time, 100% (5 × 10E4) AGC, 45 s dynamic exclusion duration with ± 10 ppm mass tolerance and exclusion lists were shared among CV’s.

### LC–MS/MS analysis for qualitative and quantitative analysis of TMT-labeled samples

For TMT-labeled peptide mixtures, we reconstituted the dried peptide fractions in 94.9:5:0.1, H2O:acetonitrile (ACN):formic acid (FA) (load solvent) and analyzed ~ 800 nanograms of each fraction by capillary LC–MS with a Thermo Fisher Scientific, Inc (Waltham, MA) Dionex UltiMate 3000 RSLCnano system on-line with an Orbitrap Eclipse mass spectrometer (Thermo Scientific, Waltham MA) with FAIMS (high-field asymmetric waveform ion mobility) separation. We injected peptides directly in load solvent and performed gradient separation on a self-packed C18 column (Dr. Maisch GmbH ReproSil-PUR 1.9 um 120 Å C18aq, 100 um ID × 40 cm length) at 55 °C with the following profile: 5% B solvent from 0 to 2 min, 8% B at 2.5 min, 21% B at 135 min, 34% B at 180 min and 90% B at 182 min with a flowrate of 325 nl/min from 0 to 2 min and 315 nl/min from 2.5 to 182 min, where solvent A was 0.1% formic acid in water and solvent B was 0.1% formic acid in ACN. The FAIMS nitrogen cooling gas setting was 5.0 L/min, the carrier gas was 4.6 L/min, and the inner and outer electrodes were set to 100 °C. We scanned the CV (compensation voltage) at −45, −60 and −70 for 1.5 s each with a data dependent acquisition method. We employed the following MS parameters: ESI voltage + 2.1 kV, ion transfer tube 275 °C; no internal calibration; Orbitrap MS1 scan 120 k resolution in profile mode from 400 – 1400 m*/z* with 50 ms injection time; 100% (4 × 10E5) automatic gain control (AGC); MS2 was triggered on precursors with 2–6 charges above 2.5E4 counts; MIPS (monoisotopic peak determination) was set to Peptide; MS2 settings (all CV’s) were: 0.7 Da quadrupole isolation window, 38% fixed collision energy, Orbitrap detection with 50 K resolution at 200 m*/z*, first mass fixed at 110 m*/z*, 150 ms max injection time, 250% (1.25 × 10E5) AGC, 30 s dynamic exclusion duration with ± 10 ppm mass tolerance and exclusion lists were shared among CV’s.

### Data analysis of TMT-labeled samples

#### Sequence database searching

We processed peptide tandem MS using SEQUEST [45] (Thermo Scientific) in Proteome Discoverer 2.5. The human Universal Proteome (UP000005640) protein sequence database was downloaded from Uniprot.org on Sept 20, 2021 and merged with a common lab contaminant protein database (https://www.thegpm.org/crap/, groups 1, 2 and 3) for a total of 78,182 total protein sequences. We applied the precursor mass recalibration node with precursor mass tolerance 20 ppm, product ion tolerance 0.1 Da, dynamic mass TMTpro (+ 304.2071 m*/z*) on K (for TMT labeled samples) and fixed carbamidomethyl (CAM) modification (+ 57.0215 m*/z*) of C. The SEQUEST database search parameters were: enzyme trypsin full specificity, 2 missed cleave sites; peptide length 6 – 50 amino acids, precursor tolerance 15 ppm, fragment ion tolerance was 0.06 Da. We specified CAM cysteine (+ 57.021 Da) as a fixed modification and the dynamic modifications were TMTpro on K and peptide N-terminus (for TMT labeled samples), acetylation of protein N-terminus (+ 42.011 Da), oxidation of M (+ 15.995 Da), conversion of Q to pyroglutamic acid (−17.027 Da), M loss at the protein N-terminus (−131.040 Da), M loss + acetylation at the protein N-terminus (−89.030 Da) and deamidation of N and Q (+ 0.984 Da). For protein inference, we applied 1% protein and peptide False Discovery Rate (FDR) filters using the Percolator algorithm [46] in PD.

#### Protein quantification

We used Proteome Discoverer (PD) for TMT-based protein quantification with the following parameters: unique and razor peptides were included, shared peptides were excluded, impurity corrections were applied, co-isolation threshold maximum was 50%, normalization was performed on the total peptide amount, protein ratio calculations were performed using pairwise ratio-based mode, which is similar to the method employed in MaxLFQ [47], and hypothesis testing was performed using the t-test. For multi-TMT experiments, we scaled the average reporter ion abundances of the pooled ‘control’ channel to 100 and scaled all other TMT reporter channels proportionally. PD employed the Benjamini–Hochberg false discovery rate procedure to control for errors associated with multiple hypothesis tests [48].

### LC–MS/MS analysis for repeatability assessment using label-free methods (see Additional file 4: Table S3)

For these experiments, a representative BALF sample was divided into three equal portions and each of these portions was processed using our workflow as described above. The resulting tryptic peptides from each of the three separate replicates were analyzed directly by LC–MS/MS analysis using two instrument platforms. For LFQ quantification, the samples were analyzed using the Orbitrap Eclipse platform, as described above for unlabeled peptide mixtures. For spectral counting quantification, analytical separation and detection of replicate mixtures were performed on an UltiMate 3000 RSLCnano UHPLC system (Thermo Scientific, Waltham, MA) interfaced to an Orbitrap Fusion Tribrid mass spectrometer (Thermo Fisher Scientific, San Jose, CA). All dried peptide samples were reconstituted using a load solvent mixture of 97.9:2:0.1, H2O:acetonitrile:formic acid. 200 nanograms of peptide mixture in 2 μL were injected on the analytical platform equipped with a 10 μL injection loop. Chromatographic separation was performed using a self-packed C18 column (Dr. Maisch GmbH ReproSil-PUR 1.9 μm 120 Å C18aq, 100 μm ID × 45 cm length) maintained at 55 °C for the duration of the experiment. The LC solvents were (A) 0.1% formic acid in H2O and (B) 0.1% formic acid in acetonitrile solutions. Chromatographic separation was performed using a linear gradient as follows: 5% B solvent from 0 to 2 min, 8% B at 2.5 min, 21% B at 40 min, 35% B at 60 min, and 90% B from 62 to 69 min followed by a return to starting conditions. The flow rate is operated at 400 nL/min for 0–2 min, 315 nL/min 2.5–60 min, and 400 nL/min for 62–69 min. A Nanospray Flex ion source (Thermo Fisher Scientific) was used with a source voltage of 2.1 kV and ion transfer tube temperature of 250 °C. Discovery LC–MS/MS analyses were performed using full-scan detection followed by data dependent MS2 acquisition (DDA). Full-scan detection was performed using Orbitrap detection at a resolution of 120,000, automatic gain control (AGC) targeted setting of 4 × 105, and a maximum ion injection time of 50 ms. Scan ranges of 380 m/z–1580 m/z were used for full-scan detection. MS2 spectra were collected using a DDA design with a 3 s cycle time in centroid mode. Fragment spectra were acquired with quadrupole isolation of 1.6 m/z, ion trap detection, and an AGC setting of 1 × 104 with a 35 ms maximum injection time. The analysis of peptides utilized CID fragmentation at a constant collision energy of 35% and 10 ms activation time.

For quantitative analysis of the replicate results, the results from the Orbitrap Eclipse analysis were analyzed by Proteome Discoverer 3.0. This LFQ analysis includes steps for feature extraction, chromatographic alignment, peptide mapping to features, protein abundance calculation, normalization, protein relative abundance ratio calculation. The results from the Orbitrap Fusion analysis were quantified using normalized spectral counting. Here, only proteins identified with at least 15 total PSMs or more summed across the replicates were considered for quantification. For each of the three samples, the spectral counts were normalized to the total number of PSMs collected for the specific replicate sample, to account for any errors due to sample loading differences. Each protein was assigned a normalized spectral count value, which was used to calculate relative abundance ratios across the three replicate samples.

## Supplementary Information

**Additional file 1: Figure S1.** Representative spectra of contamination results; precipitation vs. S-traps.

**Additional file 2: Table S1.** Protein and peptide yield at each step of the BALF processing workflow for 45 individual BALF samples.

**Additional file 3: Table S2.** Complete qualitative peptide/protein identifications from a TMT16 plex and quantitative analysis results of the TMT-labeled repeatability experiment.

**Additional file 4: Table S3.** Complete listing of peptides and inferred proteins identified from replicate experiments and quantitative analysis results from repeatability experiment using LFQ and spectral counts.

**Additional file 5: Table S4.** Quantitative protein and peptide identification across one TMT 16-plex group, comparing microfractionated vs. unfractionated analysis.

## Data Availability

The mass spectrometry proteomics datasets generated and analyzed during the current study have been deposited to the ProteomeXchange Consortium via the PRIDE [49] partner repository with the dataset identifier PXD038522. . The mass spectrometry proteomics datasets generated and analyzed during the current study are available in the Zenodo repository with a data set identifier (DOI) https://doi.org/10.5281/zenodo.7688661. The data can be accessed via https://doi.org/10.5281/zenodo.7688661.
